# Decoupling between the response of coral calcifying fluid pH and calcification to ocean acidification

**DOI:** 10.1038/s41598-017-08003-z

**Published:** 2017-08-08

**Authors:** S. Comeau, C. E. Cornwall, M. T. McCulloch

**Affiliations:** 1 The University of Western Australia, School of Earth Sciences and Oceans Institute, Crawley, Western Australia 6009 Australia; 2 ARC Centre of Excellence for Coral Reef Studies, Crawley, Western Australia 6009 Australia

## Abstract

Evaluating the factors responsible for differing species-specific sensitivities to declining seawater pH is central to understanding the mechanisms via which ocean acidification (OA) affects coral calcification. We report here the results of an experiment comparing the responses of the coral *Acropora yongei* and *Pocillopora damicornis* to differing pH levels (8.09, 7.81, and 7.63) over an 8-week period. Calcification of *A*. *youngei* was reduced by 35% at pH 7.63, while calcification of *P*. *damicornis* was unaffected. The pH in the calcifying fluid (pH_cf_) was determined using δ^11^B systematics, and for both species pH_cf_ declined slightly with seawater pH, with the decrease being more pronounced in *P*. *damicornis*. The dissolved inorganic carbon concentration at the site of calcification (DIC_cf_) was estimated using geochemical proxies (B/Ca and δ^11^B) and found to be double that of seawater DIC, and increased in both species as seawater pH decreased. As a consequence, the decline of the saturation state at the site of calcification (Ω_cf_) with OA was partially moderated by the DIC_cf_ increase. These results highlight that while pH_cf_, DIC_cf_ and Ω_cf_ are important in the mineralization process, some corals are able to maintain their calcification rates despite shifts in their calcifying fluid carbonate chemistry.

## Introduction

Ocean acidification (OA) results from a shift in seawater carbonate chemistry due to the uptake of anthropogenic CO_2_ by the oceans, and has been identified as a major threat for calcifying organisms^1^. Of particular concern are the effects of OA on scleractinian corals that form calcium carbonate reefs, a major ‘hotspot’ for marine biodiversity^2^. It has been extensively demonstrated, particularly in laboratory-based studies^3, 4^, that there is generally a negative effect of OA on coral calcification rates. However, recent studies indicate more nuanced responses to OA. For example, some species, such as massive *Porites* spp., are particularly tolerant to OA both in the laboratory^5, 6^ and in field studies^7^. Such species-specific responses have led to the hypothesis that ocean acidification could lead to a major shift in species composition between a small group of ecological winners and a larger group of ecological losers^7^.

It is therefore important to determine whether the susceptibility of corals to OA is determined by specific physiological traits, such as their morphology, rate of calcification^6, 8^, or their capacity to maintain high pH at the site of calcification when seawater pH declines^9, 10^. Maintaining elevated pH in the extracellular calcifying fluid, where precipitation of calcium carbonate occurs, is a critical step to initiate and sustain the mineralization process^9^. Direct measurements of pH in the calcifying fluid (pH_cf_) using pH microelectrodes, confocal microscopy, and isotopic methods using the boron isotope proxy have all shown that corals actively maintain pH_cf_ well above seawater pH^9, 11–13^. It has also been shown under laboratory conditions that pH_cf_ tends to partially follow changes in external pH, maintaining an offset between pH_cf_ and external seawater pH even under acidified pH conditions^9, 12, 13^. Such capacity to maintain elevated pH_cf_ could be one of the reasons explaining the resistance of some corals to modifications of the carbonate chemistry^10^. In addition to maintenance of pH at the site of calcification, physiological traits such as skeleton morphology or absolute rate of calcification have also been suggested as playing an important role in controlling the sensitivity of corals to OA^6, 14^. The rate of calcification in particular could be a key parameter, with fast calcifiers generally being more sensitive to OA than slow calcifiers, thus providing a possible explanation for the species-specific^6^ and intraspecific^14^ sensitivities to OA. While there are examples of slow calcifiers being affected by OA^6^, it is possible that the greater sensitivity of fast calcifiers can be explained by their greater requirement to export larger quantities of protons (H^+^) from their site of calcification. This requirement arises from the reaction: Ca^2+^ + HCO_3_^−^ → CaCO_3_ + H^+^ and the closely associated need to maintain a chemical micro-environment favoring mineralization through high pH (>8.6)^12^ and elevated CaCO_3_ saturation state (Ω)^9, 15^.

With increasing OA, the concentration of protons in seawater increases, which effectively steepens the proton concentration gradient between the site of calcification and the surrounding seawater. As a result, the energy required to transport protons across this larger gradient will also increase, potentially representing a significant additional metabolic cost for calcification as OA increases^16^. However it has also been questioned whether the maintenance of elevated pH and the export of protons against the proton concentration gradient is in fact an energetically expensive processes relative to the available energy resources^9^.

In addition to the maintenance of elevated levels of calcifying fluid pH_cf_, corals also have to maintain adequate supplies of dissolved inorganic carbon (DIC) and calcium at a sufficient level to elevate the aragonite saturation state in the calcifying fluid (Ω_cf_) to facilitate calcification. While several methods now exist to measure pH_cf_, which all provide similar results, measurements of the DIC concentration in the calcifying fluid (DIC_cf_) has proved much more challenging. For example, DIC_cf_ derived from micro-sensors measurements^17^ of carbonate-ion concentration was interpreted as being similar to seawater DIC levels. However, these results were highly spatially dependent and could not be determined in conjunction with pH_cf_ measured at the same location. In contrast, geochemical proxies^18, 19^ and modelling of aragonite saturation at the site of calcification^9, 20^ both indicate that DIC_cf_ is approximately double than DIC in seawater. As pH_cf_ and DIC_cf_ have a direct effect on the chemical conditions (Ω_cf_) in the calcifying fluid, measuring these two parameters in parallel is a critical step when assessing the tolerance of coral calcification to OA.

The present study was designed to test two main hypotheses regarding the response of corals to OA. First, we tested whether a rapidly calcifying coral^6^ was more sensitive to OA than a slow calcifying coral from the same location. Second, we tested whether a coral species that has been identified as being particularly tolerant to OA (*P*. *damicornis*)^21^ was able to maintain constant carbonate chemistry conditions in its calcifying fluid independently of the surrounding seawater conditions. To test these hypotheses, we designed an experiment in which one coral species potentially sensitive to ocean acidification (*Acropora yongei*) and one expected to be resistant (*Pocillopora damicornis*) were incubated over an 8-week period under three different levels of seawater pH (~8.1, 7.8, and 7.6). We measured calcification rates from 36 individuals for the two species grown under these different pH conditions and determined both the pH_cf_ and DIC_cf_ (calculated from estimates of [CO_3_^2−^]_cf_ and pH_cf_) using both isotopic (δ^11^B) and geochemical (B/Ca) proxies (see following).

## Materials and Methods

### Coral collection

The experiment was performed from October to December 2015 at the Indian Ocean Marine Research Centre’s new mesocosm facility at Watermans Bay, Western Australia, Australia. Corals used during the experiments consisted of 36 × 5-cm branches of *Acropora yongei* and 36 × 5-cm branches of *Pocillopora damicornis* that were collected 15 days prior to the beginning of the experiment from Salmon Bay, Rottnest Island, Western Australia, at ~1–2-m depth. Rottnest Island is located ~10 km offshore from Watermans Bay. Therefore, the translocated specimens were subjected to initially very similar conditions. After 2 days of recovery in continuously renewed seawater, the branches were glued to plastic bases (4 × 4 cm) with Z-Spar (A788 epoxy) to form nubbins. Nubbins were placed in the experimental tanks and allowed to recover from translocation and to acclimate to the laboratory conditions over a further 12 days.

### Treatments and regulation of pCO_2_

Seawater pCO_2_ was manipulated in twelve 20 L header tanks in which seawater was continuously renewed at ~0.3 L min^−1^. Seawater was pumped from 12-m depth, 150-m offshore, in Waterman Bay and filtered over series of sand filters (corresponding to a mesh size of 20 μm) before delivery to the header tanks. Each header tank supplied via gravity three incubation containers (for a total of 36 incubation containers) in which individual organisms for each species were maintained, along with two individual coralline algae used in a separate experiment^22^. Light was provided by 150 W LED (Malibu LED, Ledzeal) that followed a natural diel cycle. Light was gradually ramped-up in the morning commencing from 6:00 h until noon to reach a maximum of ~400 μmol quanta m^−2^ s^−1^ at noon, and then ramped down until total darkness at 18:30 h. Temperature was kept at 21 °C, which is the average seawater temperature in Salmon Bay during the austral spring^23^. The total alkalinity levels were monitored periodically and were found to be relatively constant (see following) due to continuous delivery of fresh seawater that prevented the tank water chemistry from being altered by the organisms’ metabolism.

The twelve header tanks were used to create three pH treatments in quadruplicate that corresponded to: (1) a present day pH (pH ~ 8.1; pCO_2_ ~ 400 μatm), (2) a pH value commonly predicted by the end of the current century under representative concentration pathway (RCP) 6.0 (pH ~ 7.8; pCO_2_ ~ 750 μatm)^24^, and (3) a pessimistic pH projection for the end of the century under RCP 8.5 (pH = 7.6; pCO_2_ ~ 1200 μatm). pH treatments were controlled in the header tanks using pH-controllers (AquaController, Neptune systems, USA). The set-point pH was determined by a feed-back mechanism that varied the rate of bubbling of pure CO_2_ in the header tanks. The typical precision on the set-point pH was 0.03 unit over a full 24 hour period. Treatment water was continuously delivered from each header tank to their respective ×3 incubation tanks at ~100 mL min^−1^. Submersible water pumps provided continuous turbulent water motion in each incubation tank.

### Carbonate chemistry

Seawater pH on the total scale (pH_T_) and temperature were measured at 09:00 h every ~2 d in each incubation tank, using a pH meter calibrated every 2 d on the total scale using Tris/HCl buffers^25^. Total alkalinity (*A*_T_) was measured weekly in the header tanks and 12 randomly chosen incubation tanks using a spectrophotometric method. *A*_T_ was calculated using a modified Gran function^25^, and titrations of certified reference materials (CRM) provided by A.G. Dickson (batch 151) yielded *A*_T_ values within 2 µmol kg^−1^ of the certified value. *A*_T_, pH_T_, temperature, and salinity were used to calculate the carbonate chemistry parameters using the seacarb package^26^ running in R software (R Foundation for Statistical Computing) (Table 1).

**Table 1:** Mean carbonate chemistry for each treatment during the 8-week experiment.

Treatment	pH_T_	*A*_T_ (μmol kg^−1^)	*C*_T_ (μmol kg^−1^)	pCO_2_ (μatm)	Ω_arag_	T (°C)
Ambient	8.09 ± 0.05	2358 ± 5	2061 ± 2	369 ± 4	3.28 ± 0.02	20.9
High	7.81 ± 0.05	2358 ± 5	2201 ± 1	779 ± 6	1.94 ± 0.01	20.9
Very High	7.63 ± 0.05	2357 ± 5	2270 ± 1	1217 ± 9	1.36 ± 0.01	21.0

The mean ± SE dissolved inorganic carbon (*C*_T_), partial pressure of CO_2_ (pCO_2_), and the saturation states of aragonite (Ω_arag_) were calculated from pH_T_, total alkalinity (*A*_T_), and temperature (T).

### Calcification rates

Prior to the incubation, the skeletons of the organisms were stained by placing the organisms for 30–60 min in seawater enriched with the fluorescent dye calcein at 50 mg L^−1^ with a pH adjusted to ~8.1. Single specimens of *P*. *damicornis* and *A*. *yongei* were placed randomly in each of the 36 incubation tanks, and calcification was measured over the 8-week period using buoyant weighing^27^. The difference in buoyant weight between the beginning and end of incubation period was converted to dry weight using the density of aragonite (2.93 g cm^−3^) and used to calculate net rate of calcification. The calcification rate was normalized to surface area of the coral tissue (mg cm^−2^ d^−1^) determined using the relationship between skeleton weight and surface area previously established for this coral species from Rottnest Island^22^.

### Determination of pH_cf_, [CO_3_^2−^]_cf_ and DIC_cf_

Determination of the calcifying fluid pH_cf_, [CO_3_^2−^]_cf_ and DIC_cf_ was undertaken for all organisms after completion of the experiment using the boron isotopic proxy method^13, 28^ and the recently developed^19, 29^ B/Ca method.

In brief, the use of the δ^11^B pH proxy is based on the assumption that of the two boron species (borate and boric), only the borate ion is incorporated into the carbonate skeleton of calcifiers precipitating aragonite. This behaviour has been confirmed from the δ^11^B isotopic systematics observed in aragonitic calcifiers^30^ and in inorganic aragonite precipitation experiments, and also by measurements made using a pH sensitive dye^12^. Therefore, the δ^11^B composition of the coral’s skeleton provides direct constraints on the pH of its calcifying fluid (pH_cf_). Measurements of the skeleton geochemistry were done on the tip of the branches (first 1–2 mm) that corresponded to material deposited during the 8-week incubation as shown by the calcein staining. The selected apical-tip portion of the skeleton was then crushed in a mortar and pestle. The samples were rinsed and bleached to remove any residual organic material. Dissolution of the sample powders was undertaken in 0.5 N HNO_3_. Once dissolved, boron ions were extracted using cation ion exchange resin^31^ with δ^11^B determined on a multicollector inductively coupled plasma mass spectrometry (NU II). Measurements of the international carbonate standard JCP-1 yielded a mean value of 24.37 ± 0.17‰ (mean ± 2 SD, n = 8), which was similar to the 24.33 ± 0.11‰ (SE) reported previously^32^.

Calculations of pH_cf_ based on δ^11^B were made using the calculations (equation 1) of Trotter *et al*.^28^:

1 $${{\rm{pH}}}_{{\rm{cf}}}={{\rm{pK}}}_{{\rm{B}}}-\,\mathrm{log}[\frac{({{\rm{\delta }}}^{11}{{\rm{B}}}_{{\rm{SW}}}-{{\rm{\delta }}}^{11}{{\rm{B}}}_{{\rm{carb}}})}{({{\rm{\alpha }}}_{({\rm{B}}3-{\rm{B}}4)}{{\rm{\delta }}}^{11}{{\rm{B}}}_{{\rm{carb}}}-{{\rm{\delta }}}^{11}{{\rm{B}}}_{{\rm{SW}}}+1000\,({{\rm{\alpha }}}_{({\rm{B}}3-{\rm{B}}4)}-1))}]$$

where: δ^11^B_sw_ represents the δ^11^B in seawater (δ^11^B_sw_ = 39.61‰)^33^ and α_(B3-B4)_ = 1.0272^34^. The dissociation constant of boric acid *pK*_B_ has a well-established value at given temperatures and salinities^35^. Because of a measurement error on δ^11^B of 0.17‰, uncertainty on pH_cf_ estimates was 0.01 units.

A recently developed method^19^, based on inorganic experiments, was utilized to calculate the [CO_3_^2−^]_cf_ and combined with the pH_cf_, the DIC at the site of calcification (DIC_cf_)^29^. For the determination of [CO_3_^2−^]_cf_, calculations were based on experiments^19^ that measured the ratio of boron to calcium in aragonite precipitated inorganically under a wide range of carbonate chemistries. These showed that the substitution of boron into aragonite is closely linked to the carbonate ion concentration^19^. Thus, in combination with δ^11^B derived pH_cf_, the B/Ca ratio provides a quantitative means to determine the [CO_3_^2−^] and hence the [DIC] of the calcifying fluid (i.e. DIC_cf_). B/Ca ratios were determined on the same aliquot of the solution used for pH_cf_ estimates, and DIC_cf_ was calculated from estimates of carbonate ion concentrations using the following equation (2):

2 $${[{{\rm{CO}}}_{3}^{2-}]}_{{\rm{cf}}}={{\rm{K}}}_{{\rm{D}}}{[{\rm{B}}{({\rm{OH}})}_{4}^{-}]}_{{\rm{cf}}}/{(B/\mathrm{Ca})}_{{{\rm{CaCO}}}_{3}}$$

where *K*_D_ = *K*_D.0_ exp(−*k*_*KD*_[H^+^]_T_) with *K*_D,0_ = 2.97 ± 0.17 × 10^−3^ (±95% CI), ${k}_{KD}$ = 0.0202 ± 0.042^29^. The concentration of DIC_cf_ was then calculated from estimates of pH_cf_ and [CO_3_^2−^]_cf_^29^.

Uncertainties on the estimates of DIC_cf_ and Ω_cf_ were calculated by using Monte-Carlo simulations that randomly used values of the determined parameters (δ^11^B and B/Ca) between the mean ± SD (δ^11^B SD = 0.17‰ and B/Ca SD = 18) over 1000 iterations.

A “bio-inorganic model” of calcification based on abiotic rate kinetics of CaCO_3_ precipitation (IpHRAC model)^9^ was calculated for the range of Ω_cf_ estimates calculated in this study and the experimental temperature. This model is based on an empirical exponential rate dependence law for carbonate precipitation (*G*): *G* = *k*_*a*_ (*Ω*_*cf*_ − 1)^*n*^, where *k*_*a*_ = −0.0177T^2^ + 1.47 T + 14.9, *n* = 0.0628 T + 0.0985, and T is the temperature.

### Statistical analysis

The assumptions of normality and equality of variance were evaluated through graphical analyses of residuals using the R software. Effects of each header tank on the response of calcification, pH_cf_ and DIC_cf_ to the CO_2_ treatments were analyzed using a two-way ANOVA, with the physiological measurement (e.g., calcification, pH_cf_, and DIC_cf_) as the dependent variable, CO_2_ treatments as fixed effect, and header tank as a random factor. When no significant effects of the header tank were detected and p > 0.25, header tank was dropped from the analysis^36^, and individual organisms were treated as statistical replicates. All statistical analyses were done with R.

## Results

Carbonate chemistry was successfully manipulated in all the treatments, with seawater pH_T_ maintained at 8.09 ± 0.05, 7.81 ± 0.05, and 7.63 ± 0.05 (mean ± SE) when grouped by pH treatments, which corresponded to pCO_2_ of 369 ± 4, 779 ± 6, and 1217 ± 9 μatm, respectively (Table 1). Hereafter these treatments will be referred to as pH 8.1, 7.8 and 7.6 for convenience. Because seawater was continuously renewed in the tanks, total alkalinity was maintained at ~2358 ± 5 μmol kg^−1^ in all treatments. At the end of the experiment all corals were alive in all treatments and no bleaching as a function of the pH was observed.

**Figure 1:**
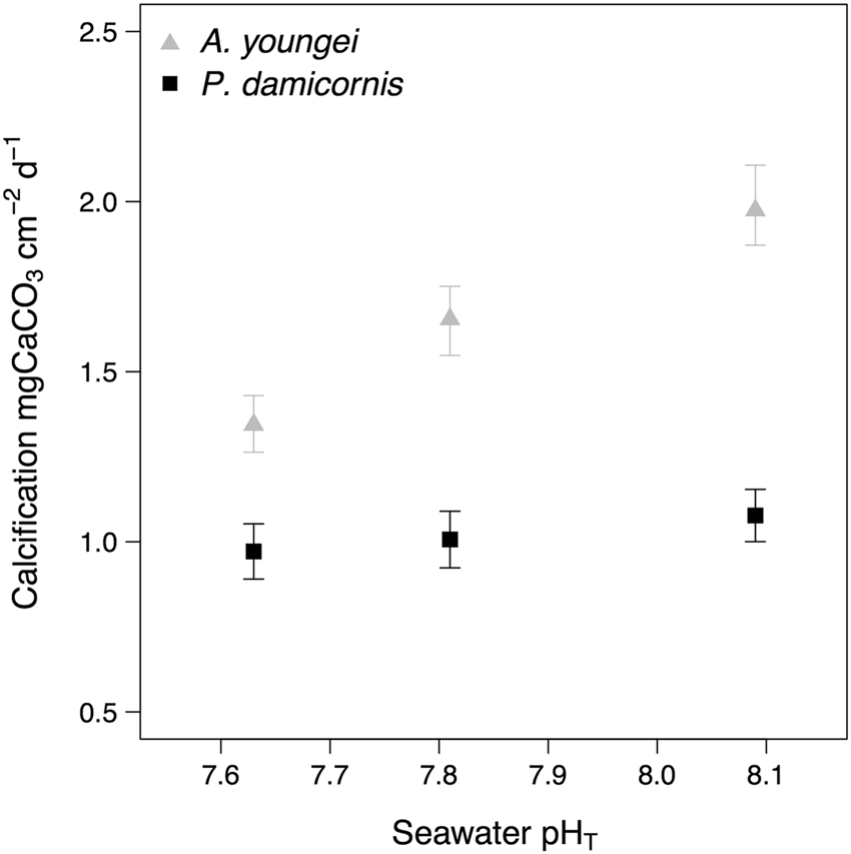
Effects of pCO_2_ on the surface area-normalized net calcification of the corals *Acropora youngei* (grey triangles) and *Pocillopora damicornis* (black dots). Calcification was measured on corals that were incubated during 8 weeks under pH = 8.09, 7.81, and 7.63. Values displayed are mean ± SE (n = 12).

For *Acropora youngei,* calcification was the highest in the pH 8.1 treatment (1.97 ± 0.13 mgCaCO_3_ cm^−2^ d^−1^) and the lowest (1.34 ± 0.09 mgCaCO_3_ cm^−2^ d^−1^) in the pH 7.6 treatment (Fig. 1A, Table 2). This indicates a significant effect of seawater pH on calcification (p < 0.001) with calcification rates decreasing as a function of decreasing pH. There was no significant effect of seawater pH on calcification for *P*. *damicornis* (*p* = 0.674) (Fig. 1B, Table 2).

**Table 2:** Mean calcification rates, pH in the calcifying fluid (pH_cf_), Dissolved Inorganic Carbon in the calcifying fluid (DIC_cf_), and aragonite saturation state in the calcifying fluid (Ω_cf_) determined on the coral *A*. *youngei* and *P*. *damicornis* incubated during 8 weeks under pH = 8.09, 7.81, and 7.63.

Species	Treatment	Calcification (mgCaCO_3_ cm^−2^ d^−1^)	pH_cf_	DIC_cf_ (μmol kg^−1^)	Ω_cf_
*A*. *youngei*	pH 8.1	1.97 ± 0.13	8.51 ± 0.01	3866 ± 47	14.0 ± 0.1
	pH 7.8	1.65 ± 0.10	8.46 ± 0.02	4044 ± 87	13.3 ± 0.2
	pH 7.6	1.34 ± 0.09	8.44 ± 0.02	4127 ± 82	13.1 ± 0.2
*P*. *damicornis*	pH 8.1	1.07 ± 0.08	8.48 ± 0.02	3493 ± 64	12.0 ± 0.3
	pH 7.8	1.00 ± 0.08	8.40 ± 0.02	3697 ± 74	10.9 ± 0.2
	pH 7.6	0.97 ± 0.08	8.35 ± 0.02	3834 ± 70	10.2 ± 0.2

Values are mean ± SE (n = 12).

**Figure 2:**
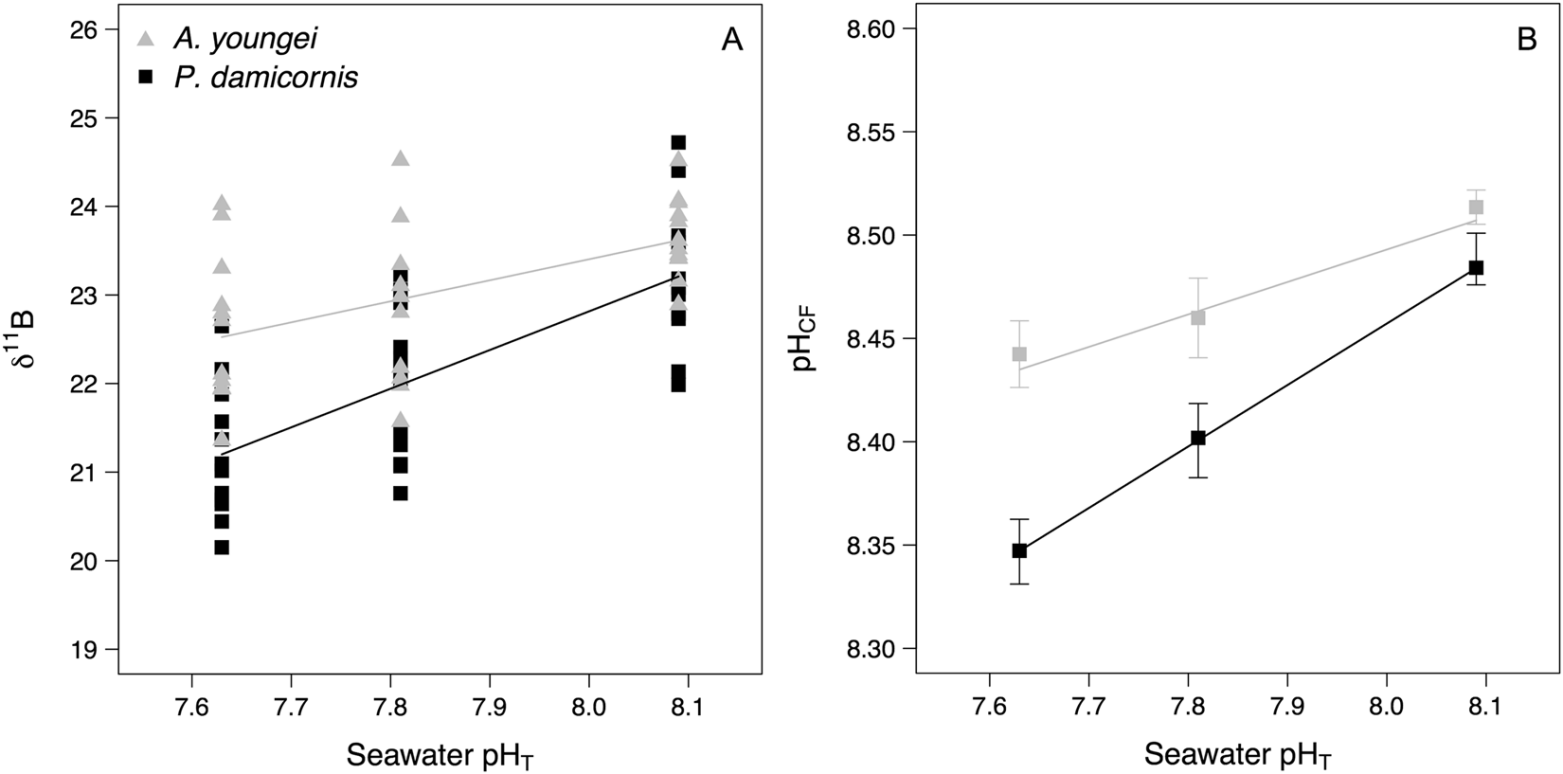
δ^11^B and estimates of pH in the calcifying fluid (pH_cf_) determined on the corals *Acropora youngei* (grey triangles) and *Pocillopora damicornis* (black dots) incubated during 8 weeks under pH = 8.09, 7.81, and 7.63. For both corals, the relationships between δ^11^B and seawater pH were best fit with linear models (δ^11^B = 2.37 pH_SW_ + 4.41, p < 0.001, and δ^11^B = 4.37 pH_SW_ − 12.11, p < 0.001, for *A*. *youngei* and *P*. *damicornis*, respectively). Therefore, the relationships between pH_cf_ and seawater pH also were best fit with linear models (pH_cf_ = 0.157 pH_SW_ + 7.24, p < 0.001, and pH_cf_ = 0.30 pH_SW_ + 6.08, p < 0.001, for *A*. *youngei* and *P*. *damicornis*, respectively). Values displayed for δ^11^B are individual replicates and are mean ± SE (n = 12) for pH_cf_. Uncertainty on pH_cf_ estimates was 0.01 pH unit.

In *A*. *youngei*, δ^11^B was significantly affected by the pH treatment (p = 0.004) and decreased linearly with decreasing seawater pH following the equation δ^11^B = 2.37 pH_SW_ + 4.41 (Fig. 2A; Raw data available in the Supplementary Table 1). pH_cf_, calculated from boron isotopes, decreased with seawater pH in *A*. *youngei* from 8.51 ± 0.01 in the ambient seawater pH (8.1) to 8.45 ± 0.02 in the pH 7.6 treatment (Fig. 2B, Table 2). Nevertheless there were small but significant effects of seawater pH on pH_cf_ (p = 0.004), and posthoc analyses showed that pH_cf_ differed between seawater pH 8.1 and pH 7.8 (TukeyHSD posthoc analyses, p = 0.044) and between seawater pH 8.1 and pH 7.6 (TukeyHSD posthoc analyses, *p* = 0.004). The linear decrease of pH_cf_ with seawater pH for *A*. *youngei* followed the equation pH_cf_ = 0.157 pH_SW_ + 7.24. For *P*. *damicornis*, seawater pH also significantly affected δ^11^B (p < 0.001) that decreased linearly with decreasing seawater pH (δ^11^B = 4.37 pH_SW_ −12.11; Fig. 2A; Raw data available in the supplementary Table 2). As a result, pH_cf_ of *P*. *damicornis* also decreased linearly with seawater pH (pH_cf_ = 0.30 pH_SW_ + 6.08; Fig. 2B, Table 2). Treatments significantly affected pH_cf_ (p < 0.001), with posthoc analyzes showing that pH_cf_ differed between all treatments (p ≤ 0.05 for all comparisons). However, there was no relationship between calcification rates and pH_cf_ for both corals (Supplementary Figure 1).

**Figure 3:**
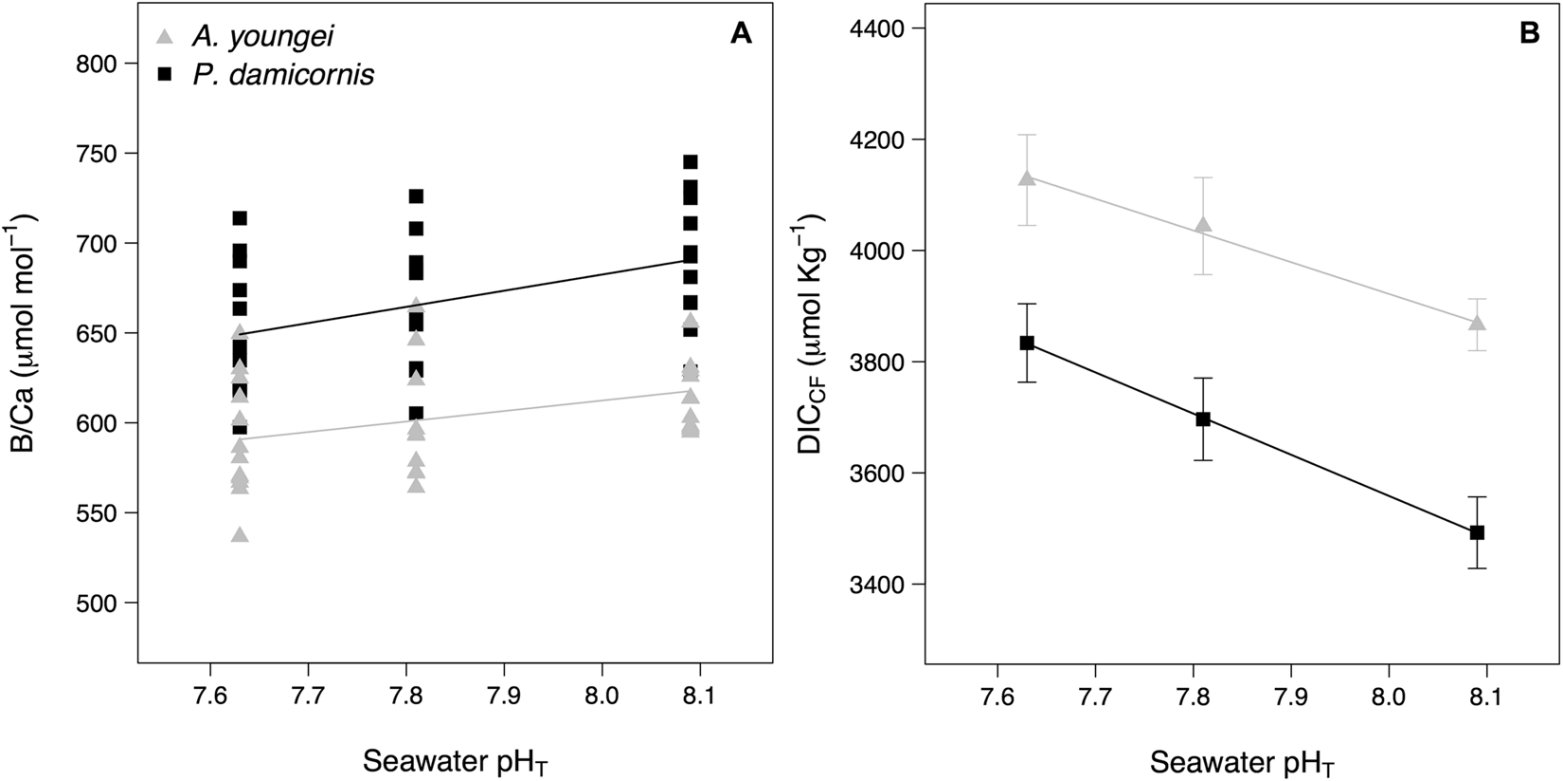
Measured B/Ca ratios (**A**) and DIC in the calcifying fluid (DIC_cf_) estimates (**B**) for the corals *Acropora youngei* (grey triangles) and *Pocillopora damicornis* (black dots). Measurements and estimates were made on corals incubated at pH = 8.09, 7.81, and 7.63 during 8 weeks. Values for the B/Ca ratios are individual measurements and displayed DIC_cf_ are mean ± SE (n = 12). Uncertainty on DIC_cf_ estimates was 110 μmol. Kg^−1^.

The B/Ca ratio varied for *A*. *pulchra* from 618 ± 6 to 591 ± 10 μmol mol^−1^ at pH 8.1 and pH 7.6, respectively, but there was no significant pH treatment effect (p = 0.101) (Fig. 3A). For *P*. *damicornis*, there was a significant pH effect on the ratio B/Ca (p = 0.036) that decreased from 690 ± 10 μmol mol^−1^ at pH 8.1 to 649 ± 11 μmol mol^−1^ at pH 7.6 (Fig. 3A). Posthoc analysis showed that the B/Ca ratio differed between pH 8.1 and pH 7.6 (TukeyHSD; p = 0.027). For both corals, the B/Ca ratios increased linearly as a function of δ^11^B (and then pH_cf_) (B/Ca = 31.6 δ^11^B–124, p < 0.001, R^2^ = 0.78, and B/Ca = 25.8 δ^11^B + 98.5, p < 0.001, R^2^ = 0.57, for *A*. *youngei* and *P*. *damicornis*, respectively; Supplementary Figure 2).

The calculated DIC_cf_ based on the combined δ^11^B and B/Ca systematics for *A*. *pulchra* ranged between 3866 ± 46 and 4127 ± 81 μmol kg^−1^ in the pH 8.1 and pH 7.6 treatments, respectively, while for *P*. *damicornis* DIC_cf_ ranged from 3493 ± 64 at pH 8.1 to 3833 ± 70 μmol kg^−1^ at pH 7.6 (Fig. 3B, Table 2). For both corals there was a significant effect of the seawater pH on DIC_cf_ (p = 0.041 and 0.005, for *A*. *pulchra* and *P*. *damiconis*, respectively).

**Figure 4:**
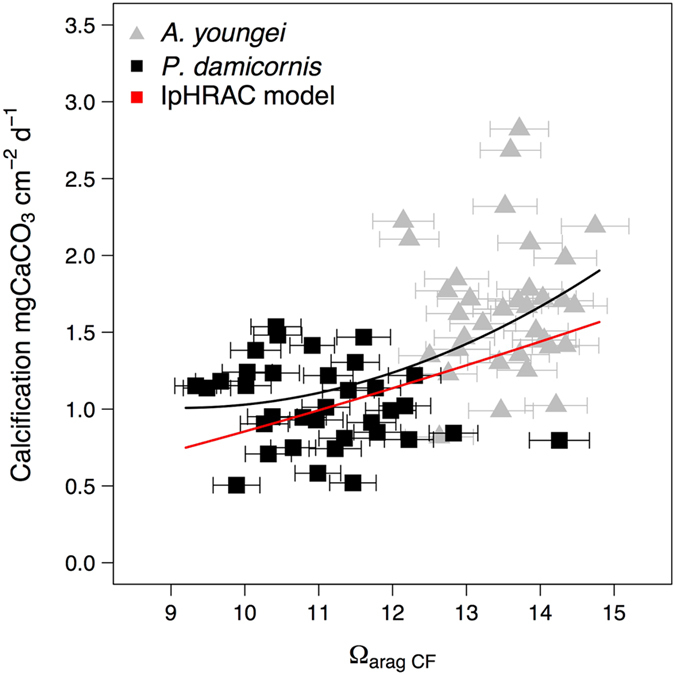
Calcification rates of *Acropora youngei* (grey triangles) and *Pocillopora damicornis* (black dots) as a function of estimates of the aragonite saturation state in the calcifying fluid (Ω_arag cf_) made using estimates of pH_cf_ and DIC_cf_. The horizontal error-bars represent the uncertainties on Ω_arag cf_ estimates. The mean relationship between calcification and Ω_aragCF_ for both species was best fitted by the polynomial relationship y = 0.025*X* ^2^ + 0.63*x* + 3.03 (black line). The red lines corresponded to a “bio-inorganic model” of calcification based on the known abiotic rate kinetics of CaCO_3_ precipitation as a function the aragonite saturation state Ω_cf_ (IpHRAC)^9^. This model is based on an empirical exponential rate dependence law for carbonate precipitation (*G*): *G* = *k*_*a*_ (*Ω*_*cf*_ − 1)^*n*^; with *k*_*a*_ = −0.0177T^2^ + 1.47 T + 14.9 and *n* = 0.0628 T + 0.0985.

Estimations of the aragonite saturation state in the calcifying fluid (Ω_cf_) from pH_cf_ and DIC_cf_ showed that it was more elevated in *A*. *pulchra* than in *P*. *damicornis* (Fig. 4, Table 2). For both species there was a significant treatment effect on Ω_cf_ (p = 0.001).

## Discussion

Understanding the role played by seawater carbonate chemistry on the chemistry of the calcifying fluid, and quantifying the physiological control of corals on their calcifying environment is of critical importance in assessing the future of coral in a high pCO_2_ world. Our study showed contrasting responses of calcification, pH_cf_, and DIC_cf_ to seawater pH in two subtropical corals from Western Australia. Calcification of the fast growing species *A*. *youngei* was more affected by OA than calcification of the slower growing *P*. *damicronis*, but pH_cf_ was more affected by seawater pH in *P*. *damicornis*. DIC_cf_ increased in both species with decreasing seawater pH, with a larger increase in *P*. *damicornis*. These results were contrary to our original hypothesis that both calcification and pH_cf_ would be more affected by OA in *A*. *youngei* than in *P*. *damicornis*. These findings were somewhat unexpected and demonstrate the complex link between calcification and carbonate chemistry within the calcifying fluid.

### Calcification

The pH treatments had a very strong species-specific effect with rates of calcification declining by 35% between pH 8.1 and pH 7.6 in *A*. *youngei*, while they did not change significantly in *P*. *damicornis*. These results are consistent with previous findings; i.e. that the calcification of *P*. *damicornis* is generally insensitive to OA across several different locations^21^ (but see also ref. 37). The tolerance of *P*. *damicornis* from Rottnest Island to OA, confirms that these inferences extends even to those living in subtropical environments. The *P*. *damicornis* specimens calcified here at rates that were similar or higher to those in past studies at 27 °C under higher light^21^ and to those recorded *in situ* ^23^. This illustrates the capacity of this species to maintain optimal growth rates under various pCO_2_, temperature and light regimes.

The genus *Acropora* is generally highly sensitive to acidification^38, 39^ (but see also refs 40, 41): this sensitivity to OA can now be extended to *A*. *youngei*. Importantly rates of calcification (~1–2 mgCaCO_3_ cm^−2^ d^−1^), and the species-specific responses to OA of these corals growing in a sub-tropical region were highly comparable to those previously reported for the same species/genus in warmer more oligotrophic waters^6^. Together, the responses of both species examined demonstrate that sub-tropical coral have similar sensitivities to OA to their tropical counterparts.

It has been suggested that for Mediterranean corals^8^ and tropical corals and calcifying algae^6, 14^ that fast calcifiers might be more sensitive to a decline in seawater pH than slow calcifiers. As previously noted this maybe due to faster rates of calcification leading to the requirement for higher rates of export of protons from the calcifying fluid, a process that may become more difficult under OA^6, 16, 42, 43^, and whose energetic requirements are still questioned^9^. In contrast, slow calcification produces fewer protons and requires the import of less carbon to the site of calcification^6^. While the present study is only based on two corals species, it tends to support this hypothesis, since the rapidly growing coral such as *A*. *youngei* are more susceptible to OA. However, the export of protons from the calcifying fluid is not the only parameter controlling calcification. This is revealed by the capacity of *P*. *damicornis* to maintain constant calcification despite the relative sensitivity of its pH_cf_ to decreasing seawater pH.

#### *pH*_*cf*_ at the site of calcification

Determining the pH_cf_ at the site of calcification is a powerful tool, providing insights into the likely mechanisms controlling the response of corals to OA. Past studies have shown that corals upregulate their pH_cf_ well above seawater pH, and that a decrease of seawater pH leads to a decrease in pH_cf_^9, 12, 13^. The present results are in agreement with these previously mentioned studies as both *A*. *youngei* and *P*. *damicornis* up-regulated their pH_cf_ well above seawater (0.43 and 0.40 pH unit in ambient conditions for *A*. *youngei* and *P*. *damicornis*, respectively), and pH_cf_ decreased significantly with decreasing seawater pH. The greater decrease in pH_cf_ (~0.3 units per seawater pH unit) with decreasing seawater pH in *P*. *damicornis* demonstrates that the magnitude of the decrease in pH_cf_ and rates of calcification are not directly correlated (Supplementary Figure 1). In contrast to *Porites cylindrical* - a coral able to maintain both calcification and pH_cf_ at ambient values when exposed to pH 0.25 units below ambient^10^ – the coral *P*. *damicornis* was able to maintain calcification at ambient values across a range of treatments despite decreasing pH_cf_.

The δ^11^B values measured here for *A*. *youngei* were within the same range as that previously reported for this genus^44–46^ (Supplementary Figure 3). However, the limited decrease of pH_cf_ (δ^11^B) with decreasing seawater pH in *A*. *youngei* (0.16 pH_cf_ unit per seawater pH unit) is unusual for species from this genus, for which a greater sensitivity of pH_cf_ to seawater pH has previously been reported (e.g., 0.51 pH_cf_ unit per seawater pH unit^9^). Different analytical methods (MC-ICPMS vs NTIMS) could explain a portion of the differences between the present study and previous measurements done on *Acropora* ^44^. However, the large differences found at low seawater pH cannot be only explained by analytical discrepancies. Furthermore, the decrease in pH_cf_ may not always be linear in *Acropora*. For example, the coral *Acropora digitifera* had a pH_cf_ that decreased rapidly between pH 8.2 and 7.8 before leveling out at pH 7.4^46^. The difference between the non-liner relationship found previously^46^, and the linear relationship found in the present study, was principally caused by higher values of δ^11^B in this previous study^46^ at control seawater pH (Supplementary Figure 3). At lower seawater pH, the δ^11^B values and the direction of the curves of the present study and that of Tanaka *et al*.^46^ were in agreement (Supplementary Figure 3). This demonstrates a very similar response of pH_cf_ to seawater pH in these two *Acropora* species.

The contradictory strong decrease in calcification and limited decrease in pH_cf_ for *A*. *youngei* can potentially be explained by two non-exclusive hypotheses. First, as daytime calcification is about ~3 fold higher than dark calcification^47^, the isotopic composition of the skeleton mostly represents conditions in the calcifying fluid during the day. In the case of *A*. *youngei*, we can hypothesize that this coral was able to maintain elevated calcification and pH_cf_ during the day, while dark calcification could have been suppressed or negative (i.e., night dissolution^48^) in the high pCO_2_ treatments. As a result, calcification could be strongly affected by pH because of decreased nighttime calcification. Under this scenario, pH_cf_ would remain unaffected, as it represents what is occurring in the light. The second potential explanation is that pH_cf_ is not the only driver of calcification in corals that varies due to external seawater carbonate chemistry. For example, in the low pH seawater, the synthesis of organic matrix^49, 50^ could have been reduced because of less energy available for this process. This would limit the ability of *A*. *youngei* to precipitate calcium carbonate, regardless of chemically favorable conditions.

Furthermore, pH_cf_ is not the only parameter governing the chemistry in the calcifying fluid. Other parameters such as calcium and DIC concentrations can affect both the saturation state and the rates of calcium carbonate precipitation. Finally, a reduction in calcification while pH_cf_ remained almost constant could demonstrate that this coral requires elevated pH_cf_ to precipitate calcium carbonate, and is not able to elevate its rate of proton export under OA; i.e., the gradient in external to internal proton concentration increases, regardless of internal pH_cf_. As a result, a reduction in calcification due to reduced proton production export, could have been the only option available for *A*. *youngei* to maintain elevated pH_cf_ and precipitation of calcium carbonate, though at a lower rate.

#### *DIC*_*cf*_ at the site of calcification

Here we provide one of the first estimations of DIC_cf_ using geochemical proxies for corals grown under a range of controlled pH conditions. In both corals, DIC_cf_ (between ~3500 and 4100 μmol kg^−1^) was well above seawater DIC (between ~2060 and 2270 μmol kg^−1^). This is similar to that estimated for *Porites* spp., using δ^11^B and B/Ca ratio^18^, and previous studies that have estimated DIC_cf_ to be about double the seawater DIC^9, 12^. Recently, McCulloch *et al*.^29^ also showed using δ^11^B and B/Ca ratio that DIC_cf_ in massive *Porites* corals varies seasonally well above seawater DIC and can reach values as high as 3.2 times the seawater DIC in summer. The agreement between these past studies and the present results lends support for the validity of our approach. Only a recent study has reported much lower estimates of DIC_cf_ using carbonate micro-probe^17^. The discrepancy between geochemical and micro-probe approaches are still unresolved, and could be due to the spatio-temporal differences between the two methods. Micro-probe methods provide spot measurements of pH and DIC at different times and location of what is inferred to be the chemical conditions in the calcifying fluid, while geochemical proxies indicate the average chemistry in the calcifying fluid responsible for skeletal formation averaged over weeks of carbonate precipitation.

DIC_cf_ increased as a function of decreasing seawater pH for both *A*. *youngei* and *P*. *damicornis*. As a result, the two species exhibited strong linear relationships between DIC_cf_ and pH_cf_, where decreasing pH_cf_ resulted in higher DIC_cf_ (Supplementary Figure 4). This is similar to what has been found at the colony level on massive *Porites* spp. where seasonal increases in DIC_cf_ were associated with decreases in pH_cf_. Increasing the import of DIC to the calcifying fluid when pH decreases might be one strategy for corals to alleviate some of the negative effects of decreasing pH. Thereby maintaining constant DIC/proton ratios in the calcifying fluid to maintain precipitation rates. Interestingly, the increase in DIC_cf_ was larger in *P*. *damicornis*, which could explain part of the capacity of this species to maintain calcification despite decreasing pH_cf_.

Increasing DIC_cf_ with OA could be the result of several processes. Firstly, with ocean acidification, more DIC is available in seawater - DIC increased by ~200 μmol kg^−1^ between pH 8.1 and pH 7.6 - which could favor the uptake of DIC if this process is substrate limited. Secondly, a reduction in calcification could lead to less DIC being consumed, therefore favoring its accumulation in the calcifying fluid if the import of DIC remain constant. This hypothesis is mostly valid for *A*. *youngei* for which calcification decreased. Thirdly, corals might actively increase the rate at which they import DIC in the calcifying fluid under OA by actively increasing the activity of their Cl-/bicarbonate transporter^51^ to increase DIC concentration in the calcifying cell or/and the calcifying fluid^52^. Finally, the increase in DIC_cf_ (and therefore total alkalinity in the calcifying fluid) with decreasing seawater pH could be the cause of the decrease in pH_cf_, because maintaining elevated pH_cf_ in a calcifying fluid with higher buffering capacity (higher TA_cf_) is chemically more challenging.

#### Ω_cf_ at the site of calcification

Estimates of the aragonite saturation state in the calcifying fluid showed that Ω_cf_ was well above Ω_arag_ in seawater, for both corals. When pooled by treatments, Ω_cf_ appeared to be more affected by pH in *P*. *damicornis* than *A*. *youngei* because of the stronger decrease in pH_cf_ for *P*. *damicornis* (Supplementary Figure 5).

When calcification rates of the two corals species determined in this study were pooled together and plotted against Ω_cf_, calcification followed a polynomial relationship with Ω_cf_ (Fig. 4) that increased with Ω_cf_. A “bio-inorganic model” of calcification based on the known abiotic rate kinetics of CaCO_3_ precipitation as a function of saturation state (IpHRAC)^9^ was also fitted using estimates of Ω_cf_ (Fig. 4, red curve). The IpHRAC model (Fig. 4) was in agreement with the data measured here. Thus, although coral calcification is a biologically mediated process that is species-specific, a simple model (IpHRAC) was able to explain most of the mean link between calcification rates and Ω_cf_.

Nevertheless, the IpHRAC model did not capture the entire complexity of the responses, as calcification tended to drop rapidly with Ω_cf_ was elevated (for Ω_cf_ > 12) but leveled out at lower Ω_cf_ (<12). This finding tends to demonstrate that the coral with low Ω_cf_ were less affected by changes in seawater pH. Potentially, this result could indicate two different and non-exclusive means of maintaining calcification in corals. For corals calcifying at low Ω_cf_, the precipitation of CaCO_3_ could be dependent on an active biological catalyzation such as the one provided by proteins from the organic matrix, which have been shown to favor precipitation of calcium carbonate at low saturation state^50^. Such corals would therefore be less sensitive to changes in their calcifying fluid chemistry. In contrast, calcification in corals with elevated Ω_cf_ could be less dependent on the activity of catalyzers and be governed by the chemical conditions in the calcifying fluid (i.e., precipitation of CaCO_3_ occurs because Ω_cf_ is elevated). As a result, calcification in such corals would be more sensitive to changes in Ω_cf_. However it is important to bear in mind that this experiment was performed on only two corals species and more experimental data where DIC_cf_ and pH_cf_ are determined at the same time on corals maintained in identical experimental conditions and exposed to various pH levels would be necessary to confirm its broad significance. Furthermore this experiment was performed under constant conditions of temperature, light, and pH, which did not capture the complexity of natural variations that affect coral physiology and coral calcifying fluid composition *in situ* ^29^.

## Conclusion

The current study shows that pH_cf_ is not the only driver of the response to OA in sub-tropical corals. Indeed, while calcification behaved as expected, with a stronger decline in *A*. *youngei* than in *P*. *damicornis* under OA, the decline in pH_cf_ under OA was more important in *P*. *damicornis*. DIC_cf_, estimated using a novel proxy, increased in both corals with pCO_2_, but this increase was not sufficient to compensate for declining pH_cf_ as shown by the decrease in Ω_cf_ in both corals. There was a non-linear relationship between Ω_cf_ and calcification suggesting that the corals with lower rates of calcification and lower Ω_cf_ will be less affected by changes in pCO_2_. Maintaining elevated Ω_cf_ allow corals to catalyze the rapid precipitation of calcium carbonate, however, the present results indicate that maintaining such high rates of precipitation will become more challenging in the future. As a result, OA could reduce the variance of growth between corals, which could potentially have ecological repercussion on coral competition^53^. Further investigations into the potential role of the relationship Ω_cf_ – calcification rates and sensitivity to OA are necessary to estimate the effects of OA on the specific composition of future coral reefs.

## Electronic supplementary material

Supplementary information
