# Supplementary material for: Decoupling between the response of coral calcifying fluid pH and calcification to ocean acidification
Source: Sci Rep. 2017 Aug 8;7:7573. doi: 10.1038/s41598-017-08003-z (PMC5548905; doi:10.1038/s41598-017-08003-z)
Supplement: Supplementary file 1 — Supplementary information [file 41598_2017_8003_MOESM1_ESM.pdf]

Decoupling between the response of coral calcifying fluid pH and calcification to ocean acidification

S. Comeau, C. E. Cornwall, M. T. McCulloch

Supplementary Figure 1: Individual calcification rates and estimates of pH in the calcifying fluid (pH_{cf}) of the corals *Acropora youngei* (grey triangles) and *Pocillopora damicornis* (black dots) incubated during 8 weeks under pH = 8.09, 7.81, and 7.63.

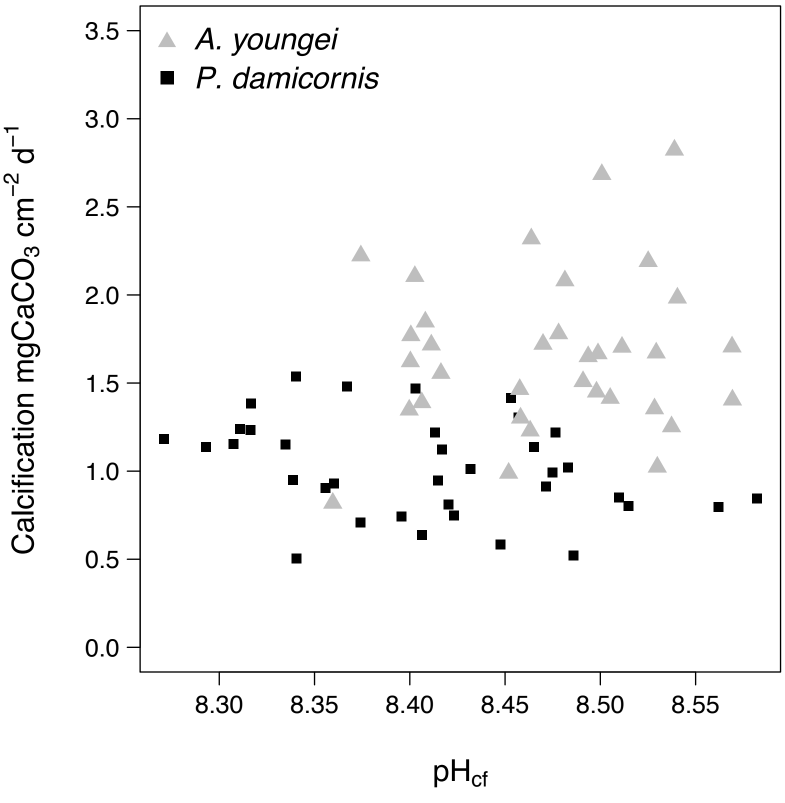

Supplementary Figure 2: Relationship between B/Ca ratios and δ^{11}B in the corals *A. pulchra* (grey triangles) and P. *damicornis* (black squares). For both corals, the relationships between B/Ca ratios and δ^{11}B were best fit with linear models (B/Ca = 31.6 δ^{11}B - 124, $p< 0.001$, $R^2 = 0.78$, and B/Ca = 25.8 δ^{11}B + 98.5, $p< 0.001$, $R^2 = 0.57$, for *A. youngei* and *P. damicornis*, respectively).

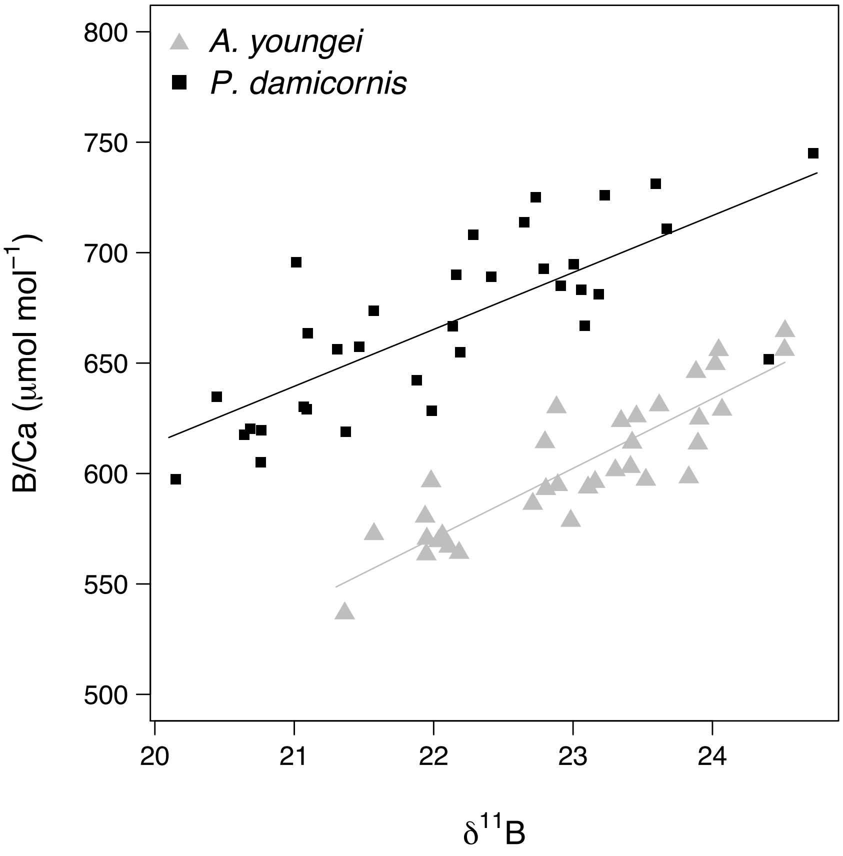

Supplementary Figure 3: Comparison of $\delta^{11}B$ values as a function of seawater pH in corals from the genus *Acropora*. $\delta^{11}B$ values are shown for the present study (grey squares), Honish et al.[41] (black circles), Reynaud et al. [42] (black triangles), and Tanaka et al.[43] (black diamonds).

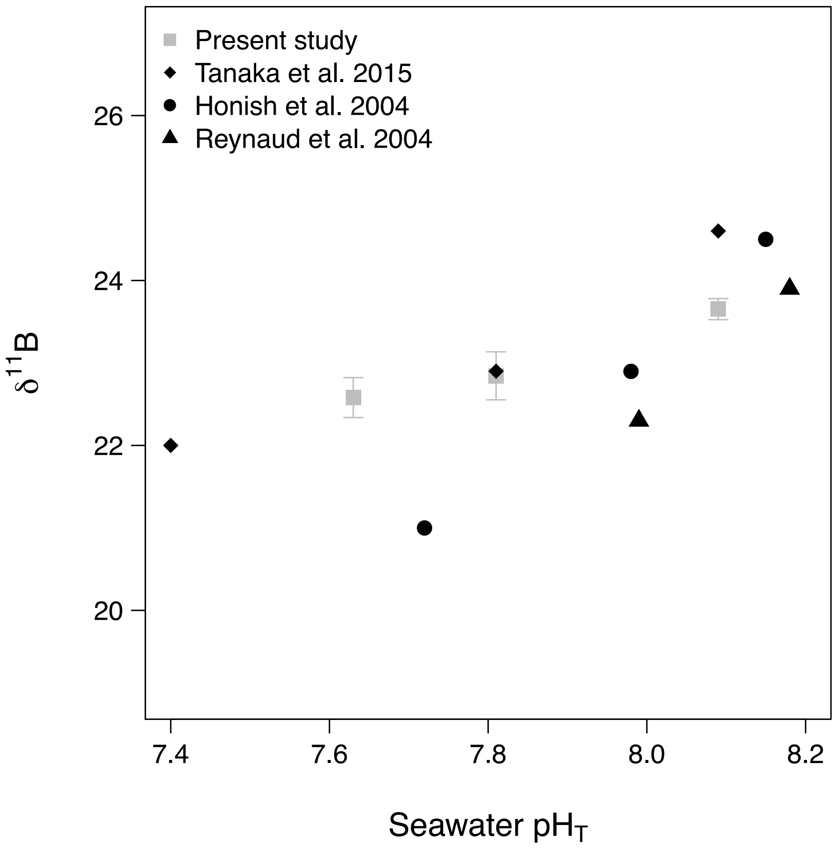

Supplementary Figure 4: Relationship between pH_{cf} and estimates of dissolved inorganic carbon concentration in the calcifying fluid (DIC_{CF}) . Measurements were done on the corals *A. pulchra* (grey triangles) and P. *damicornis* (black squares).

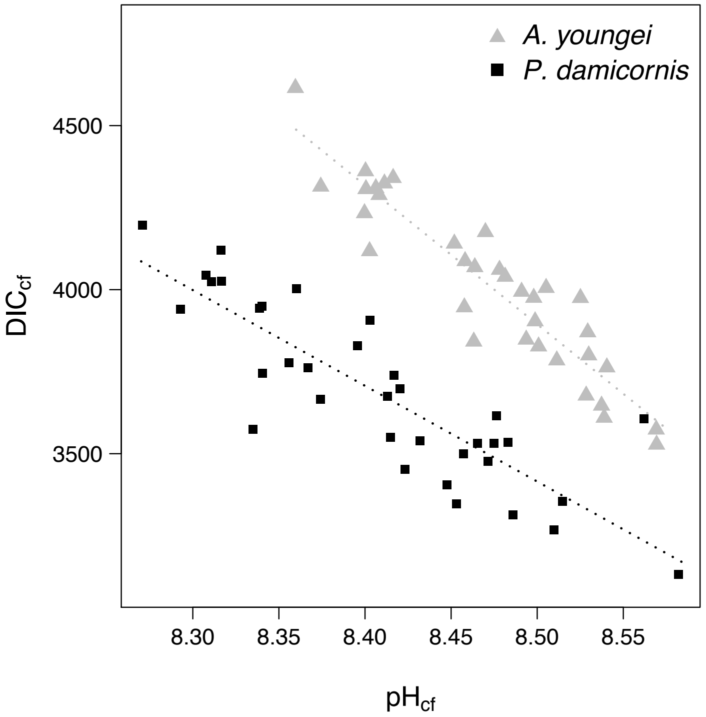

Supplementary Figure 5: Relationship between pH_{cf} and estimates of aragonite saturation state in the calcifying fluid (Ω_{CF}) . Measurements were done on the corals *A. pulchra* (grey triangles) and P. *damicornis* (black squares).

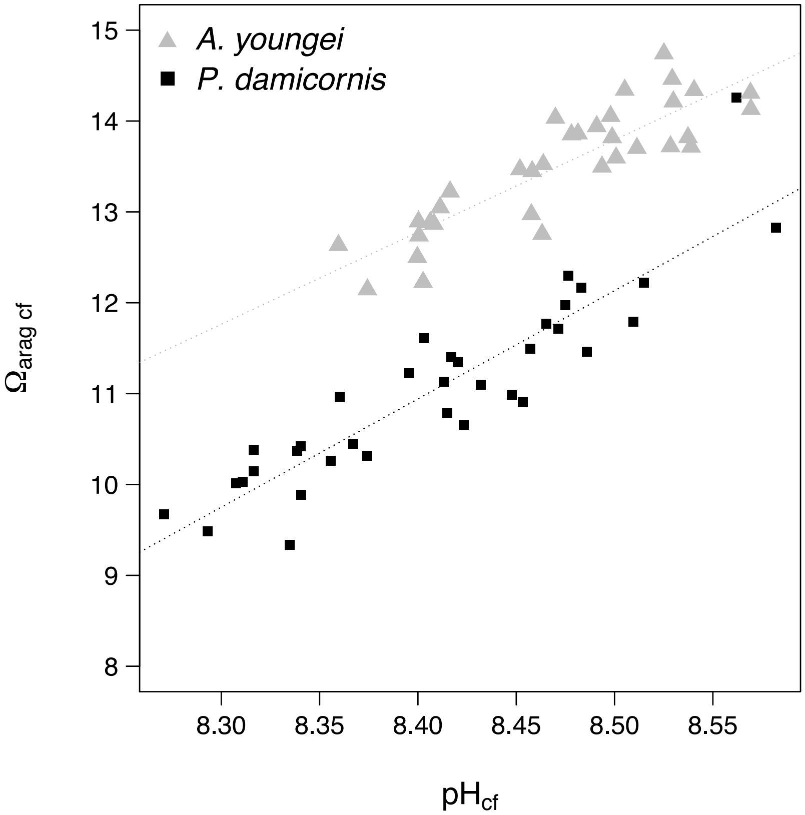

Supplementary Table 1: Individuals measurements of $\delta^{11}B$ and B/Ca made on the corals *A. youngei* incubated for 8 weeks under pH 8.1, 7.8 and 7.6.

Species	Treatment	$\delta^{11}B$	pH_{cf}	B/Ca	DIC_{cf}	Calcification
A. youngei	8.1	23.16	8.48	596.2	4040	2.08
	8.1	23.83	8.53	598.3	3975	2.19
	8.1	23.9	8.53	613.6	3870	1.67
	8.1	22.89	8.46	594.8	4069	2.32
	8.1	23.52	8.51	597.2	4006	1.41
	8.1	23.41	8.50	603	3976	1.45
	8.1	24.07	8.54	628.9	3763	1.98
	8.1	24.04	8.54	655.9	3610	2.82
	8.1	23.42	8.50	614	3904	1.67
	8.1	23.62	8.51	630.9	3785	1.70
	8.1	24.52	8.57	656	3574	1.71
	8.1	24.06	8.54	653	3625	2.32
	7.8	22.98	8.47	578.6	4176	1.72
	7.8	22.18	8.42	564.1	4341	1.56
	7.8	22.06	8.41	572	4289	1.85
	7.8	23.11	8.48	593.7	4061	1.78
	7.8	22.81	8.46	593	4087	1.30
	7.8	23.3	8.49	623.5	3853	0.76
	7.8	22.31	8.42	617.9	3955	1.03
	7.8	21.98	8.40	596.5	4118	2.11
	7.8	23.34	8.49	623.8	3848	1.65
	7.8	24.52	8.57	664.5	3528	1.40
	7.8	21.57	8.37	572.6	4314	2.22
	7.8	23.88	8.53	646	3678	1.35
	7.6	21.36	8.36	536.7	4615	0.82
	7.6	21.94	8.40	580.5	4233	1.35
	7.6	22.71	8.45	586.3	4141	0.99
	7.6	23.9	8.53	624.9	3800	1.02
	7.6	22.11	8.41	566.9	4324	1.72
	7.6	23.3	8.49	601.5	3994	1.51
	7.6	21.95	8.40	563.4	4361	1.62
	7.6	22.03	8.41	569.4	4310	1.39
	7.6	22.88	8.46	630	3842	1.23
	7.6	24.02	8.54	649.6	3647	1.25
	7.6	21.95	8.40	570.5	4307	1.77
	7.6	22.8	8.46	614.2	3947	1.46

Supplementary Table 2: Individuals measurements of $\delta^{11}B$ and B/Ca made on the corals *P. damicornis* incubated for 8 weeks under pH 8.1, 7.8 and 7.6.

Species	Treatment	$\delta^{11}B$	pH_{cf}	B/Ca	DIC_{cf}	Calcification
P. damicornis	8.1	22.73	8.45	725.1	3347	1.41
	8.1	22.14	8.41	666.7	3675	1.22
	8.1	22.79	8.46	692.6	3500	1.30
	8.1	23.01	8.47	694.8	3476	0.91
	8.1	23.18	8.48	681.1	3535	1.02
	8.1	24.40	8.56	651.8	3606	0.80
	8.1	23.67	8.51	710.9	3356	0.80
	8.1	23.59	8.51	731.2	3267	0.85
	8.1	24.72	8.58	745.1	3133	0.84
	8.1	21.99	8.40	628.5	3907	1.47
	8.1	23.08	8.48	667	3616	1.22
	7.8	22.41	8.43	689.2	3540	1.01
	7.8	21.07	8.34	630.3	3944	0.95
	7.8	21.09	8.34	629.1	3950	1.54
	7.8	22.19	8.42	654.8	3739	1.12
	7.8	21.31	8.36	656.3	3777	0.90
	7.8	21.47	8.37	657.4	3763	1.48
	7.8	22.28	8.42	708	3453	0.75
	7.8	23.23	8.49	726	3313	0.52
	7.8	22.91	8.47	685	3532	1.14
	7.8	23.06	8.47	683.3	3531	0.99
	7.8	20.76	8.32	605.2	4121	1.23
	7.8	22.24	8.42	661.4	3699	0.81
	7.6	20.76	8.32	619.5	4026	1.38
	7.6	20.64	8.31	617.5	4044	1.15
	7.6	21.02	8.33	695.7	3575	1.15
	7.6	21.57	8.37	673.8	3666	0.71
	7.6	21.10	8.34	663.5	3745	0.50
	7.6	20.44	8.29	634.7	3941	1.14
	7.6	21.88	8.40	642.3	3829	0.74
	7.6	22.65	8.45	713.8	3405	0.58
	7.6	22.16	8.41	689.9	3550	0.95
	7.6	21.37	8.36	618.8	4002	0.93
	7.6	20.69	8.31	620.2	4024	1.24
	7.6	20.15	8.27	597.5	4196	1.18
